# Automatic Detection and Classification of Knee Osteoarthritis Using Hu's Invariant Moments

**DOI:** 10.3389/frobt.2020.591827

**Published:** 2020-11-16

**Authors:** Shivanand S. Gornale, Pooja U. Patravali, Prakash S. Hiremath

**Affiliations:** ^1^Department of Computer Science, Rani Channamma University, Belagavi, India; ^2^Department of Master of Computer Application (MCA), Karnataka Lingayat Education Society (KLE) Technological University, Hubballi, India

**Keywords:** knee radiography, osteoarthritis (OA), KL grading, Hu's invariant moments, K-NN

## Abstract

Significant information extraction from the images that are geometrically distorted or transformed is mainstream procedure in image processing. It becomes difficult to retrieve the relevant region when the images get distorted by some geometric deformation. Hu's moments are helpful in extracting information from such distorted images due to their unique invariance property. This work focuses on early detection and gradation of Knee Osteoarthritis utilizing Hu's invariant moments to understand the geometric transformation of the cartilage region in Knee X-ray images. The seven invariant moments are computed for the rotated version of the test image. The results demonstrated are found to be more competitive and promising, which are validated by ortho surgeons and rheumatologists.

## Introduction

**Figure 1:**
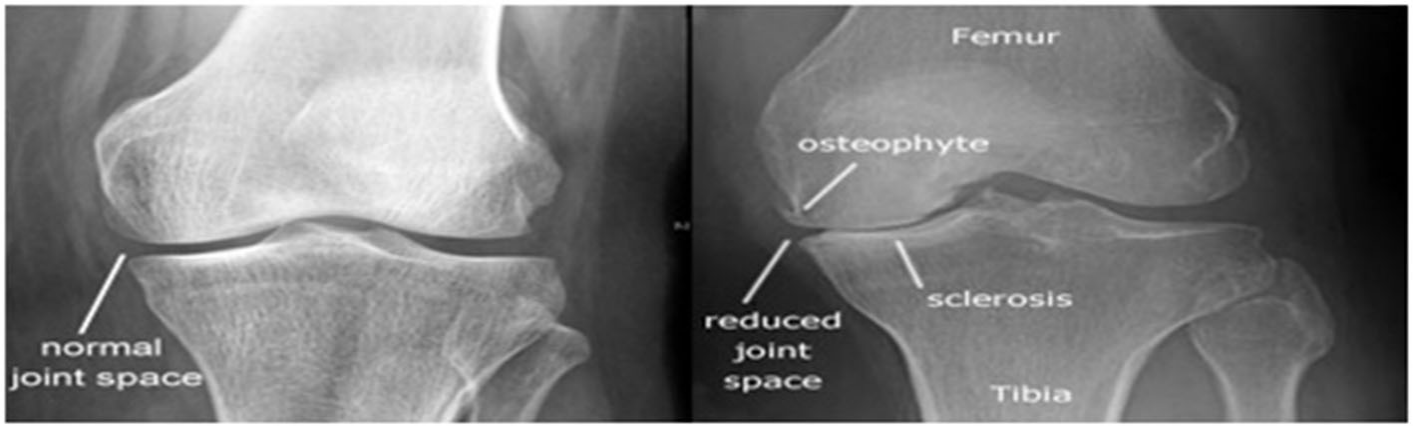
Radiological parameters of Knee Osteoarthritis (Stemcellsdoc's Weblog, 2011).

Knee Osteoarthritis (OA) is a human knee joint condition that primarily impacts cartilage. Cartilage has a significant role to perform in leg movement. In OA, the top layer of cartilage disintegrates and deteriorates resulting in intense pain (Gornale et al., 2020c). The patient suffering from knee pain has to consult a doctor, who will then test the clinical symptoms of the patient and advise the patient to go for radiographic imaging of the knee. Clinical symptoms play a crucial role in the treatment of osteoarthritis (de Graaff et al., 2015). The proper analysis of the illness is carried out by observing both clinical signs and radiological criteria. Relevant radiological parameters are width of the joint area, osteophytes, sclerosis, etc. The important radiological parameters are depicted in Figure 1. Based on the radiological parameters, the severity level of the ailment is analyzed using the Kellgren and Lawrence (KL) grading system (Reichmann et al., 2011; Gornale and Patravali, 2017). The KL system is the most common method used to classify the knee joint OA into 5 different grades essentially to discern the severity of the disease (Kellgren and Lawrence, 1957; Stemcellsdoc's Weblog, 2011). Knee radiographic images are very sensitive to unintended defects that may create complications in the study of bone structures. It may result in the experts taking more time to analyse the Knee x-ray and infer the existence of OA. Thus, in order to overcome these problems, this work focuses on early detection and gradation of Knee OA utilizing Hu's invariant moments to understand the geometric transformation of the cartilage region in Knee X-ray images.

## Related Work

In this work, an extensive survey has been carried out, which indicates that many researchers have primarily worked on different segmentation techniques for extracting the cartilage region either automatically or semi automatically. While other researchers have focused solely on various feature extraction methods by manually extracting the region of interest, minimal experiments have been performed in the evaluation of ailments as per the Kellgren and Lawrence grading system. The findings presented in the literature are considered to be satisfactory. But there is still room to create a more important and reliable automatic computer aided algorithm for OA detection that will contend with state-of-the-art techniques.

Gornale et al. (2016b,c, 2017, 2019a,b,c, 2020a,b) have used the semi-automated active contour method for the extraction of the cartilage region and experimented with their own database of 500 Knee X-ray images. The different statistical features, geometric features and Zernike moments are computed and classified obtaining the accuracy of 87.92% for random forest classifier and 88.88% for K-NN classifier (Gornale et al., 2016b,c). Subsequently, in the next experiment with the extended dataset, the gradient features are computed and classified obtaining an accuracy of 95% (Gornale et al., 2017). Further, different segmentation methods are implemented for the extraction of cartilage. The basic mathematical features are computed and classified giving an accuracy of 97.55% (Gornale et al., 2019a). Further, a novel approach to identify and extract the region of interest based on the density of pixels is implemented in (Gornale et al., 2020a). The extracted region is then used for computation using a histogram of the oriented gradient method and local binary pattern, the experimentation being done on a dataset of 1,173 Knee X-ray images. The results demonstrated an accuracy of 97.86 and 97.16% with respect to Medical Expert-I and Medical Expert-II opinions. In (Gornale et al., 2019b), with an expanded dataset of 1,650 knee X-ray images, an algorithm that calculates the cartilage thickness/area has been developed. The calculated thickness demonstrated an accuracy of 99.81% using a K-NN classifier, which is evaluated by radiographic expert incompliance with KL grading framework. In order to provide improved performance for the denser regions, local phase quantization and projection profile features are computed and classified. The gradation was done using Artificial Neural Network and an accuracy of 98.7 and 98.2% as per the Expert I and II opinions, respectively, has been achieved (Gornale et al., 2019c). Lastly, the experiments utilizing multi-resolution wavelet filters with varying filter orders and decomposition levels have been performed and classification is done using decision tree classifier. Classification accuracy of 98.54 and 97.93% as per the Expert I and II opinions is obtained for the Biorthogonal 1.5 wavelet filter at 4th decomposition level (Gornale et al., 2020b).

According to the earlier work, the key sources of distortions or noises in knee X-ray images are due to film processing and digitization. It becomes difficult to extract the significant regions from the X-ray images that are geometrically distorted. Hence, it is proposed to use Hu's invariant moments in the present analysis to address these issues as these are invariant to scale, translation and orientation.

## Material and Methods

**Figure 2:**
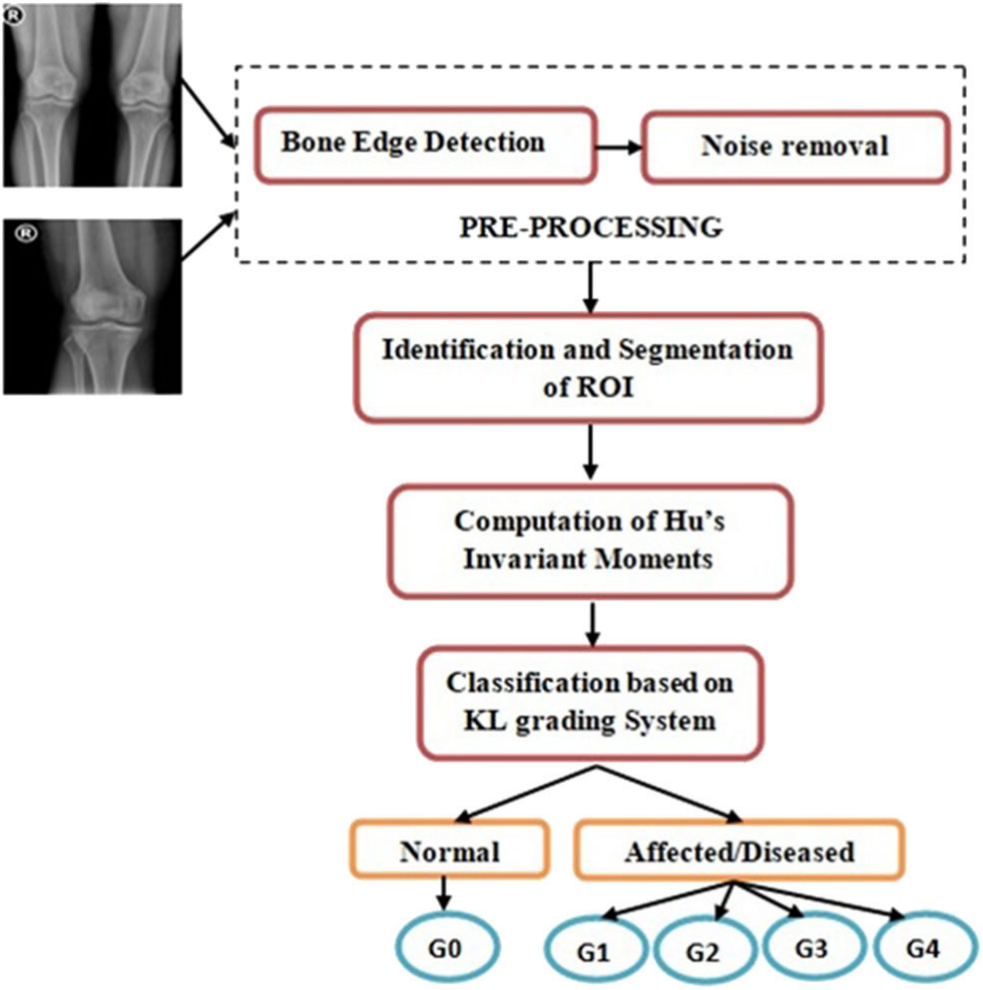
Flow diagram (G−0, Normal; G−1, Doubtful; G−2, Mild; G−3, Moderate; G−4, Severe).

The proposed method includes pre-processing that automatically detects the contours of knee bones and eliminates unwanted distortions. Then the cartilage region is identified and extracted. Features, namely, Hu's invariant moments, are computed and then classified to determine OA grading. The flow diagram is represented in Figure 2.

### Dataset

The own dataset of 2000 digital knee X-ray images collected from well-reputed local hospitals and health centers was considered for the experimentation. The fixed-flexion digital knee X-rays have been acquired using a PROTEC PRS-500E X-ray machine. Original images were 8-bit 1,350 × 2,455 grayscale images. For the experimentation, the manual annotation of these images was done by two medical experts for the gradation of each Knee X-ray according to the KL grading framework, which is summarized in the Table 1. This data is publicly available and can be found in Gornale and Patravali (2020).

**Table 1:** Manual gradation done by two medical experts, namely, Expert I and II.

**KL grade**	**Medical expert-I**	**Medical expert-II**
Grade-Normal	651	628
Grade-Doubtful	528	551
Grade-Mild	279	279
Grade-Moderate	260	260
Grade-Severe	282	282
Total	2,000	2,000

### Pre-processing

In the initial pre-processing of a knee X-ray image, the contours of knee bone are detected, which is followed by filtering to eliminate the noise content without hindering the essential information of the image (Hall et al., 1971).

### Identification and Segmentation of ROI

The cartilage region in the knee joint is the region of interest (ROI). Identification of ROI is done on the basis of pixel density, as the bone in the X-ray is denser, which results in a higher number of pixel values (Semmlow, 2004; Nithya and Santhi, 2017). Later, the detected ROI is cropped and then used as one of the inputs to the active contour algorithm for segmentation. The portion of the knee is dynamically segmented using 3 × 3 masks (Caselles et al., 1995). The object boundaries are further located iteratively by using this mask (Gornale et al., 2016b,c; Gornale et al., 2017, 2019b).

### Feature Extraction

Hu's invariant moments are computed from the segmented region for the detection and classification of Knee Osteoarthritis. Based on algebraic invariants, one skew orthogonal invariant and six orthogonal invariants were derived by Hu that are independent to geometric transformations and parallel projection (Hu, 1962). Herein, the basic principle is to depict the objects by means of measurable quantities called invariants which have adequate power of classification to differentiate between objects belonging to different classes (Li, 2010; Urooj and Singh, 2016). For a 2-D continuous function f(x,y), the moment of order (p+q) is defined in the Equation (1):

(1) $$\begin{array}{l} m_{pq}=\overset{\infty }{\underset{-\infty }{\int }}\overset{\infty }{\underset{-\infty }{\int }}x^{p}y^{q}f\left(x,y\right)dxdy \end{array}$$

where, p, q = 0,1,2,…. and f(x,y) is a piecewise continuous function that has non-zero values only in a finite portion of the xy-plane; moments of all orders arise and the moment series (m_pq_) is uniquely defined by f(x,y) (Gonzalez and Woods, 2002; Li, 2010). Conversely, m_pq_ uniquely determines f(x,y). The central moments are defined in the Equation (2):

(2) $$\begin{array}{l} \mu_{pq}=\overset{\infty }{\underset{-\infty }{\int }}\overset{\infty }{\underset{-\infty }{\int }}{\left(x-\bar{x}\right)}^{p}{\left(y-\bar{y}\right)}^{q}f\left(x,y\right)dxdy \end{array}$$

where, $\bar{x}=\frac{m_{10}}{m_{00}}$ and $\bar{y}=\frac{m_{01}}{m_{00}}$, *m*_00_ is the mass of the image.

m_10_/m_00_ and m_01_/m_00_ are the centroids of the image. The scale invariance is obtained by dividing the central moments by a proper normalization factor which is a non-zero quantity for all the test images (Hu, 1962; Huang and Leng, 2010). It is evident that the lower order moments are more robust to noise and easy to calculate (Li, 2010). The normalized central moments denoted η_pq_ are defined in the Equation (3):

(3) $$\begin{array}{l} \eta_{pq}=\frac{\mu_{pq}}{{\mu^{\gamma }}_{00}} \end{array}$$

where, $\gamma =\frac{p+q}{2}+1$, for p+q=2,3….

In 1962, Hu proposed the following seven invariant moments for in-plane rotation (Hu, 1962; Gonzalez and Woods, 2002; Li, 2010):

$$\begin{array}{l} \underset{1}{\phi }=\eta_{20}+\eta_{02}\\ \underset{2}{\phi }={\left(\eta_{20}-\eta_{02}\right)}^{2}+4\eta_{11}^{2}\\ \underset{3}{\phi }={\left(\eta_{30}-3\eta_{12}\right)}^{2}+{\left(3\eta_{21}-\eta_{03}\right)}^{2}\\ \underset{4}{\phi }={\left(\eta_{30}+\eta_{12}\right)}^{2}+{\left(\eta_{21}+\eta_{03}\right)}^{2}\\ \underset{5}{\phi }=(\eta_{30}-3\eta_{12})(\eta_{30}+\eta_{12})[{\left(\eta_{30}+\eta_{12}\right)}^{2}\\ -3{\left(\eta_{21}+\eta_{03}\right)}^{2}]+(3\eta_{21}-\eta_{03})(\eta_{21}+\eta_{03})\\ \left[3{\left(\eta_{30}+\eta_{12}\right)}^{2}-{\left(\eta_{21}+\eta_{03}\right)}^{2}\right]\\ \underset{6}{\phi }=(\eta_{20}-\eta_{02})[{\left(\eta_{30}+\eta_{12}\right)}^{2}-{\left(\eta_{21}+\eta_{03}\right)}^{2}]\\ +4\eta_{11}(\eta_{30}+\eta_{12})(\eta_{21}+\eta_{03})\\ \underset{7}{\phi }=(3\eta_{21}-\eta_{03})(\eta_{30}+\eta_{12})[{\left(\eta_{30}+\eta_{12}\right)}^{2}-3{\left(\eta_{21}+\eta_{03}\right)}^{2}]\\ +(3\eta_{12}-\eta_{30})(\eta_{21}+\eta_{03})[3{\left(\eta_{30}+\eta_{12}\right)}^{2}-{\left(\eta_{21}+\eta_{03}\right)}^{2}] \end{array}$$

### Classification

The Hu's moments computed for the segmented regions are classified using two different classifiers, namely, K-NN and Decision Tree. The analysis of results shows that the K-NN classifier produced superior results relative to decision tree classifier. The K-NN categorizes class labels depending upon the gap ratio between the evaluation data and the testing data. The relevant K value is considered by the K-NN classifier to provide a class label for unlabelled image by identifying the nearest neighbor (Gornale et al., 2016a, 2020c).

## Results

The experiment was carried out on a test image that was rotated by 15 to 90 degrees with 15-degree increments. Moments were determined for the rotated version of the test image. The findings demonstrated that the rotational invariant moments were in reasonable agreement with the invariants computed for the original image. Further, to check the robustness and the invariance, the experiment was extended to 180 degrees and it was noticed that comparable findings were obtained for all the seven invariant features. The results for rotations up to 90^0^ are shown in Table 2. The experiment is then performed to check the invariant moment for scale invariance. The test image is scaled by positive scale factor, namely, 0.2, 0.3, 0.4, 0.5, and 0.6. Hu's invariant moments are determined for various scales and the corresponding results are shown in Table 3.

**Table 2:** Moment invariants computed for varying rotation angles.

**Invariant**	**Rotated 15^**0**^**	**Rotated 30^**0**^**	**Rotated 45^**0**^**	**Rotated 60^**0**^**	**Rotated 75^**0**^**	**Rotated 90^**0**^**
Φ_1_	0.632661	0.630683	0.635225	0.63062	0.631871	0.631925
Φ_2_	0.30043	0.29898	0.303868	0.298513	0.299343	0.299581
Φ_3_	0.053643	0.052936	0.053955	0.053022	0.054119	0.054094
Φ_4_	0.006241	0.005974	0.006225	0.006154	0.006227	0.006191
Φ_5_	−0.00013108	−0.00013508	−0.00014181	−0.00013528	−0.00014673	−0.00015073
Φ_6_	−0.00235	−0.00243	−0.00244	−0.00235	−0.00242	−0.00244
Φ_7_	0.000106371	0.000106100	0.00010587	0.00010534	0.00010610	0.00010618

**Table 3:** Moment invariants for varying scales.

**Invariant**	**0.2**	**0.3**	**0.4**	**0.5**	**0.6**
Φ_1_	0.8659	0.8701	0.8653	0.8749	0.8631
Φ_2_	1.0720	1.0700	1.0701	1.0713	1.0701
Φ_3_	1.1050	1.1051	1.1053	1.1052	1.1053
Φ_4_	0.01030	0.01031	0.01029	0.01031	0.01030
Φ_5_	−0.00001684	−0.00001679	−0.00001680	−0.00001678	−0.00001680
Φ_6_	−0.0114	−0.0114	−0.0113	−0.0114	−0.0113
Φ_7_	−0.00007717	−0.00007807	−0.00007728	−0.00007710	−0.00007724

To further examine the optimality, the experimentation is carried for the entire dataset using 2-fold cross validation strategy. The classification accuracies of Decision tree and K-NN classifiers are summarized in Table 4, which reveals that the performance of the K-NN classifier is better relative to that of the decision tree classifier. Therefore, the confusion matrix of the K-NN classifier related to Expert I and II is provided in Tables 5, 6. From Table 5, it is found that the accuracy rates of 99.53% for Normal grade, 99.81% for Doubtful grade and 100% for Mild, Moderate and Severe grade were obtained. Correspondingly, from the Table 6, it is found that the accuracy rates of 99.04% for Normal grade, 96.18% for Doubtful grade and 100% for Mild, Moderate and Severe grade were obtained. The overall accuracies of 99.80% as per medical Expert-I opinion and 98.65% as per medical Expert-II were attained.

**Table 4:** Accuracies obtained by using KNN and decision tree classifiers as per Expert I and II opinion.

**Classifiers**	**Medical expert-I opinion ***%*****	**Medical expert-II opinion ***%*****
K-NN	**99.80**	**98.65**
Decision tree	95.75	95.4

*K-NN classifier accuracies are better compared to decision tree classifier so the values of K-NN are in bold*.

**Table 5:** Confusion matrix obtained by K-NN classifier as per medical Expert-I opinion.

**Class**	**Normal**	**Doubtful**	**Mild**	**Moderate**	**Severe**
Grade-Normal	**648**	1	0	0	0
Grade-Doubtful	3	**527**	0	0	0
Grade-Mild	0	0	**279**	0	0
Grade-Moderate	0	0	0	**260**	0
Grade-Severe	0	0	0	0	**282**

*The bold values are correctly classified values*.

**Table 6:** Confusion matrix obtained by K-NN classifier as per medical Expert-II opinion.

**Class**	**Normal**	**Doubtful**	**Mild**	**Moderate**	**Severe**
Grade-Normal	**622**	21	0	0	0
Grade-Doubtful	6	**530**	0	0	0
Grade-Mild	0	0	**279**	0	0
Grade-Moderate	0	0	0	**260**	0
Grade-Severe	0	0	0	0	**282**

*The bold values are correctly classified values*.

To assess the classification accuracies and the efficiency, Precision and Recall are used which are defined in Equations (4, 5).

(4) $$\begin{array}{l} \mathrm{Pr}ecision=\frac{TP}{TP+FP} \end{array}$$

(5) Recall=TPTP+FN

**Figure 3:**
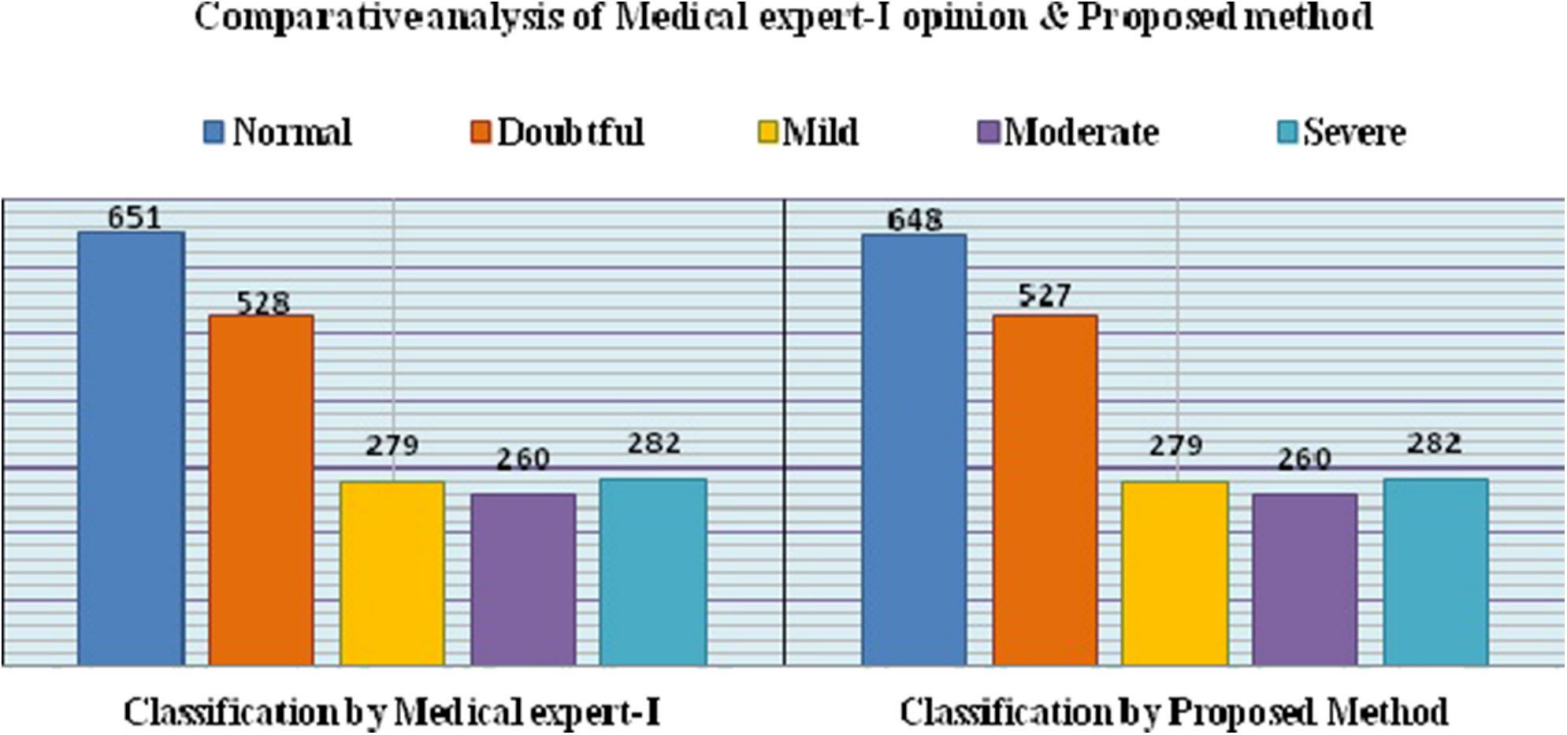
Graphical representation of medical Expert-I opinion and proposed method.

**Figure 4:**
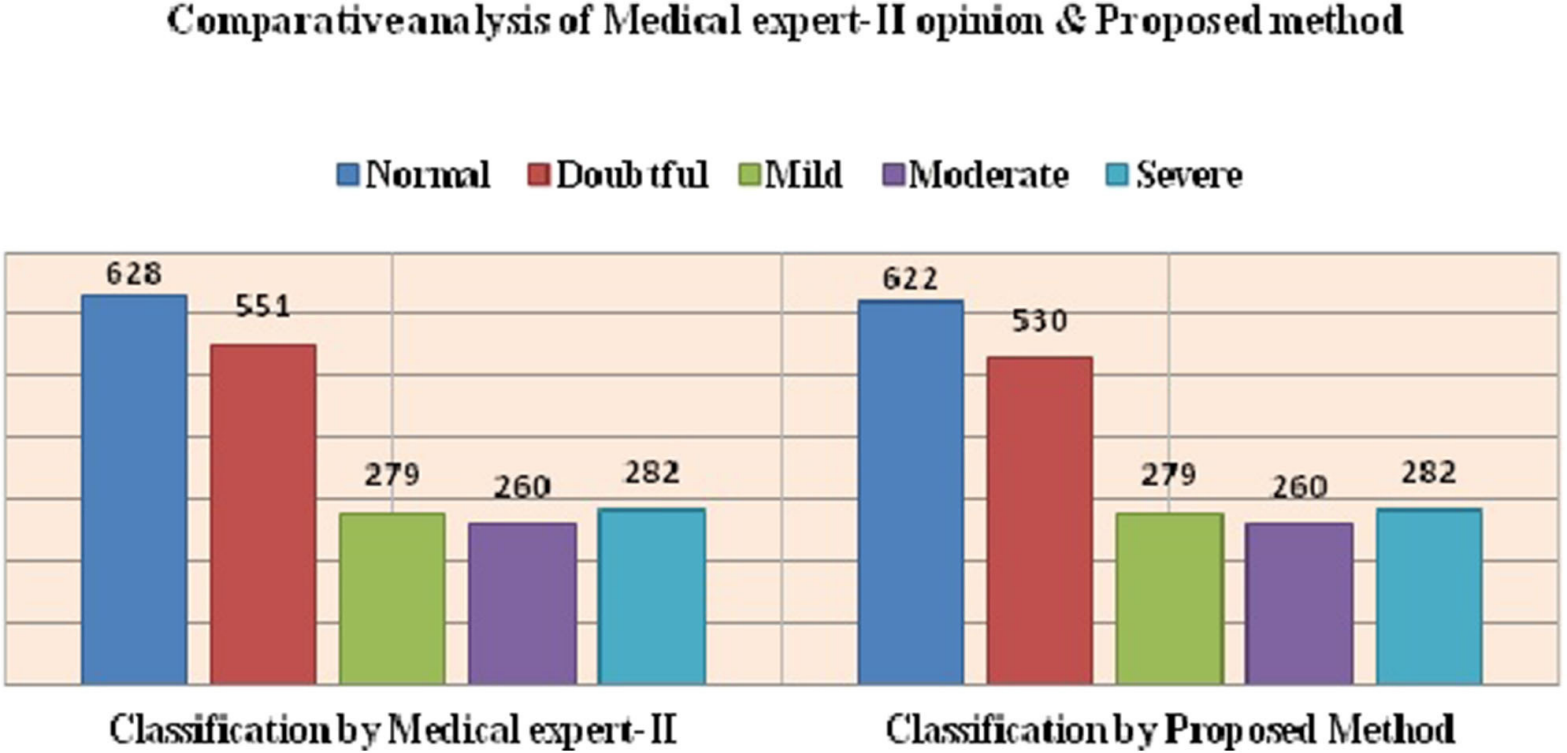
Graphical Representation of medical Expert-II opinion and proposed method.

Where, TP denotes True Positive, FP denotes False Positive and FN denotes False Negative. The precision and recall for Expert I and II opinions are given in Tables 7, 8, respectively. The comparative analysis of the results of the proposed method and Medical experts' opinions is graphically depicted in Figures 3, 4, respectively.

**Table 7:** Precision and recall as per medical Expert-I.

	**Normal**	**Doubtful**	**Mild**	**Moderate**	**Severe**
Precision	0.9954	0.9981	1	1	1
Recall	0.9985	0.9943	1	1	1

**Table 8:** Precision and recall as per medical Expert-II.

	**Normal**	**Doubtful**	**Mild**	**Moderate**	**Severe**
Precision	0.9904	0.9619	1	1	1
Recall	0.9673	0.9888	1	1	1

### Statistical Test of Significance

The significance test is performed to determine whether the outcomes of the experiments are statistically significant or not. Two different tests, namely, the Chi-square test and *t*-test, are performed to verify the inferences of the present work.

### Chi-Square Test

Chi-Square test examines whether there is relationship between two variables or not. In this test a null hypothesis is about a difference between the opinions of two medical experts. The evaluation is carried out with the findings of the proposed algorithm and the manual analysis made by medical experts. The hypotheses for Chi-Square test, performed separately in relation to medical Expert I and II, are stated as below:

- Null Hypothesis (H0): There is close consensus between the outcomes of the algorithm and the annotations made by the medical Expert I/ Expert II.
- Alternative Hypothesis (H1): No association between the findings of proposed algorithm and the annotations made by the medical Expert-I/ Expert- II
- Degree of freedom (df) = 4, the critical value of χ^2^ with df = 4 at 5% if significance level is 9.48773 (from the Chi-square table).
- If χ^2^ <9.48773, Accept H0 and reject H1 else vice versa.

The details of the Chi-square test related to medical Expert-I and II are presented in the Tables 9, 10. The computed values of the Chi-square variables in cases of the Expert I and II are given below:

$$\begin{array}{l} Expert-I-chi-square\left(\chi^{2}\right)=\sum \frac{{\left(f_{o}-f_{e}\right)}^{2}}{f_{e}}=0.006858\\ Expert-II-chi-square\left(\chi^{2}\right)=\sum \frac{{\left(f_{o}-f_{e}\right)}^{2}}{f_{e}}=0.384018 \end{array}$$

**Table 9:** Chi-square test between the results of proposed algorithm and annotations by medical Expert-I.

**Class**	**Proposed algorithm (PA)**	**Medical expert-I (ME-I)**	**Total (PA+ME-I)**	**Expected values of PA**	**Expected values of ME-I**
Normal	649	651	1,300	650	650
Doubtful	530	528	1,058	529	529
Mild	279	279	558	279	279
Moderate	260	260	520	260	260
Severe	282	282	564	282	282
Total	2,000	2,000	4,000		

**Table 10:** Chi-square test between the results of proposed algorithm and annotations by medical Expert-II.

**Class**	**Proposed algorithm (PA)**	**Medical expert-II (ME-II)**	**Total (PA+ME-II)**	**Expected values of PA**	**Expected values of ME-II**
Normal	643	628	1,271	635.5	635.5
Doubtful	536	551	1,087	543.5	543.5
Mild	279	279	558	279	279
Moderate	260	260	520	260	260
Severe	282	282	564	282	282
Total	2,000	2,000	4,000		

The computed Chi-square values are observed to be lower than the critical values obtained from the Chi-square table. Hence, the null hypothesis is accepted and the alternative hypothesis is rejected. It indicates that there is significant agreement between the findings of the proposed method and the annotations provided by both the medical experts.

### *t*-Test

The *t*-test is meant to answer the question of whether two groups are statistically different from each other. In this test a null hypothesis is about a difference between the opinions of two medical experts. The common hypotheses for all the experiments utilizing *t*-test are stated as below:

- Null Hypothesis (H0): Difference in opinions is due to random variations in samples and not due to experts.
- Alternative Hypothesis (H1): Difference in opinions is due to medical experts and not due to random variations in samples.
- Degree of freedom (df) = 4, the critical value of t with df = 4 at 5% significance level is 2.1318 (from the t distribution table).
- If t-score>2.1318, Reject H0 and accept H1 else vice versa.

The details of t-test related to medical Expert-I and II are presented in the Table 11.

$$\begin{array}{l} t-score=\frac{\sum D/N}{\sqrt{\frac{\sum D^{2}-\left(\sum D^{2}/N\right)}{\left(N-1\right)N}}}=0 \end{array}$$

Therefore, the computed *t*-value is less than the table value, and hence the hypothesis is accepted. It means that the disparity of opinions between the two experts is attributed to random variations of samples and not due to experts. Both the tests of statistical significance have demonstrated that the proposed algorithm is substantially successful and thus gives reliable computer aided assistance to physicians for rapid evaluation of the OA ailment. It helps patients to secure appropriate and reliable timely diagnosis and treatment.

**Table 11:** The *t*-test between the annotations made by medical Expert-I and II.

**Class**	**Annotations by medical expert-I (X)**	**Annotations by medical expert-II (Y)**	**D = (X–Y)**	**D^**2**^ = (X–Y)^**2**^**
Normal	651	628	23	529
Doubtful	528	551	−23	529
Mild	279	279	0	0
Moderate	260	260	0	0
Severe	282	282	0	0
		Total	ΣD=0	ΣD^2^=1,058

## Discussion

The prime cause for geometric distortions of cartilage region in knee X-ray images is the progression of OA, which could be misrepresented due to filming, handling, and digitization during image acquisition. It may become difficult in extracting the significant regions from such distorted images. Hu's invariant moments provide suitable invariant features from such distorted images due to their rotation, scale, and translation invariance properties. In this work, feature extraction using Hu's invariant moments is done for the detection and classification of Knee Osteoarthritis. The experimental results obtained for the rotated and scaled images are in reasonable agreement with the invariants computed for the original image. Overall, the proposed algorithm results, which are validated by ortho-surgeons and rheumatologists, are found to be more competitive and promising.

## Data Availability Statement

This data is already publicly available and can be found in (Gornale and Patravali, 2020).

## Ethics Statement

Written informed consent was obtained from the individual(s) for the publication of any potentially identifiable images or data included in this article.

## Author Contributions

SG: paper writing and proof reading. PP: data collection and experimental analysis. PH: testing and validation. All authors contributed to the article and approved the submitted version.

## Conflict of Interest

The authors declare that the research was conducted in the absence of any commercial or financial relationships that could be construed as a potential conflict of interest.
